# Inhibition of Serine Protease Activity Protects Against High Fat Diet-Induced Inflammation and Insulin Resistance

**DOI:** 10.1038/s41598-020-58361-4

**Published:** 2020-02-03

**Authors:** Chin-Sung Kuo, Jia-Shiong Chen, Liang-Yu Lin, Geert W. Schmid-Schönbein, Shu Chien, Po-Hsun Huang, Jaw-Wen Chen, Shing-Jong Lin

**Affiliations:** 1 Division of Endocrinology and Metabolism, Department of Medicine, Taipei Veterans General Hospital and Institute of Clinical Medicine, National Yang-Ming University, Taipei, Taiwan; 2 Institute of Clinical Medicine, National Yang-Ming University, Taipei, Taiwan; 3 Division of Endocrinology and Metabolism, Department of Medicine, Taipei Veterans General Hospital and National Yang-Ming University, Taipei, Taiwan; 4 The Institute of Engineering in Medicine, University of California San Diego La Jolla, California, US; 5 Departments of Bioengineering, Nanoengineering, Institute of Engineering in Medicine, University of California San Diego La Jolla, California, US; 6 Division of Cardiology, Department of Critical Care Medicine, Taipei Veterans General Hospital and Institute of Clinical Medicine, and Cardiovascular Research Center, National Yang-Ming University, Taipei, Taiwan; 7 Department of Medical Research and Education, Taipei Veterans General Hospital, Institute and Department of Pharmacology, and Cardiovascular Research Center, National Yang-Ming University, Taipei, Taiwan; 8 Healthcare and Services Center, and Department of Medical Research, Taipei Veterans General Hospital, Division of Cardiology, Heart Center, Cheng-Hsin General Hospital, Institute of Clinical Medicine, and Cardiovascular Research Center, National Yang-Ming University, and Taipei Heart Institute, Taipei Medical University, Taipei, Taiwan

**Keywords:** Type 2 diabetes, Obesity

## Abstract

Recent evidence suggests that enhanced protease-mediated inflammation may promote insulin resistance and result in diabetes. This study tested the hypothesis that serine protease plays a pivotal role in type 2 diabetes, and inhibition of serine protease activity prevents hyperglycemia in diabetic animals by modulating insulin signaling pathway. We conducted a single-center, cross-sectional study with 30 healthy controls and 57 patients with type 2 diabetes to compare plasma protease activities and inflammation marker between groups. Correlations of plasma total and serine protease activities with variables were calculated. In an *in-vivo* study, LDLR^−/−^ mice were divided into normal chow diet, high-fat diet (HFD), and HFD with selective serine protease inhibition groups to examine the differences of obesity, blood glucose level, insulin resistance and serine protease activity among groups. Compared with controls, diabetic patients had significantly increased plasma total protease, serine protease activities, and also elevated inflammatory cytokines. Plasma serine protease activity was positively correlated with body mass index, hemoglobin A1c, homeostasis model assessment-insulin resistance index (HOMA-IR), tumor necrosis factor-α, and negatively with adiponectin concentration. In the animal study, administration of HFD progressively increased body weight, fasting glucose level, HOMA-IR, and upregulated serine protease activity. Furthermore, *in-vivo* serine protease inhibition significantly suppressed systemic inflammation, reduced fasting glucose level, and improved insulin resistance, and these effects probably mediated by modulating insulin receptor and cytokine expression in visceral adipose tissue. Our findings support the serine protease may play an important role in type 2 diabetes and suggest a rationale for a therapeutic strategy targeting serine protease for clinical prevention of type 2 diabetes.

## Introduction

Metabolic syndrome is a global public health issue of increasing magnitude. Obesity, insulin resistance, proinflammatory and prothrombotic states, atherogenic dyslipidemia, and higher blood pressure are risk factors for metabolic syndrome^1^. Among these factors, obesity-induced insulin resistance is a major contributor to the worldwide prevalence of type 2 diabetes. Although previous reports have suggested that obesity is correlated closely with insulin resistance^1^, the molecular mechanism involved in the development of obesity and related complications remains largely unclear. A large body of evidence suggests that obesity is associated with chronic adipose tissue inflammation, which may result in enhanced proinflammatory cytokine levels^2^. Obesity has been associated not only with larger adipocytes but also with increasing numbers of infiltrating macrophages in adipose tissue^3,4^. These macrophages are currently suggested as a significant cause of obesity-associated chronic low-grade inflammation via the secretion of a wide variety of inflammatory molecules^5^.

Elevation of plasma protease activity was shown to be associated with diet-induced obesity and insulin resistance or chronic inflammation^3–9^. Badeanlou *et al*. reported that diet-induced insulin resistance required protease-activated receptor 2 signaling activation^3^. Talukdar *et al*. demonstrated that the activation of neutrophil-derived elastase (a serine protease) is involved in high-fat diet (HFD)-induced insulin resistance, which suggests that proteases play a crucial role in obesity-induced insulin resistance and type 2 diabetes^4^. Obesity has also been found to increase neutrophil-derived elastase activity and decrease the serum level of α1-antitrypsin (an endogenous serine protease inhibitor) in mice and humans^5^. α1-Antitrypsin is associated with type 2 diabetes, as 50% of diabetic patients have low circulating α1-antitrypsin levels^6,7^. Furthermore, α1-antitrypsin has been shown to prevent type 1 diabetes development, prolong islet allograft survival, and inhibit pancreatic β-cell apoptosis *in vivo*^8^. Chronic inflammation-induced proteolytic matrix metalloproteinases cleave the extracellular domains of insulin receptors, induce soluble insulin receptor-α in human plasma, and promote insulin resistance^9^. Interestingly, the recent evidence showed that the soluble insulin-receptor ectodomain is elevated in the plasma of patients with diabetes^10^, and the serine protease granzyme B is an inflammatory marker related to insulin receptor cleavage in human obesity and type 2 diabetes mellitus^11^. Accordingly, protease activity inhibition is thus a potential therapeutic target to improve impaired metabolism and insulin resistance in obese subjects.

The present study is aimed to test the hypothesis that serine protease plays a pivotal role in patients with type 2 diabetes. Low-density lipoprotein (LDL) receptor knockout (LDLR^−/−^) mice with HFD has been used as the diet-induced obesity and diabetes mouse model^12^. We investigated the postulated mechanisms that elevated plasma serine protease activities which may cause excessive proteolytic cleavage of insulin receptor-α followed by insulin resistance and related abnormal metabolic parameters in this mouse model. Furthermore, we explored the possible therapeutic effects of 4-(2-aminoethyl) benzenesulfonyl fluoride hydrochloride (AEBSF), a selective serine protease inhibitor.

## Results

### Baseline characteristics of human study

Table 1 summarizes the demographic and clinical characteristics of the human study subjects. As expected, diabetic patients had significantly higher fasting glucose and hemoglobin A1c (HbA1c) levels, waist circumference, and body mass index (BMI) than did patients without diabetes. Compared with the non-diabetic group, patients with diabetes had elevated levels of inflammatory markers, including IL-6 and high-sensitivity C-reactive protein (hs-CRP), and decreased adiponectin levels. There was no difference in lipid profiles, including total cholesterol, LDL cholesterol (LDL-c), and triglycerides between groups, except that patients with diabetes had lower high-density lipoprotein cholesterol (HDL-c) levels.

**Table 1:** Basic characteristics of study subjects.

	No diabetes (*n* = 30)	Type 2 diabetes (*n* = 57)	*p*
Age (years)	47.8 ± 12.9	53.3 ± 11.5	0.05
Male	16 (53.3%)	37 (64.9%)	0.293
Hyperlipidemia	7 (23.3%)	27 (47.4%)	0.029
Hypertension	5 (16.7%)	21(36.8%)	0.051
Coronary artery disease	2 (6.7%)	7 (12.3%)	0.713
Current smoker	0 (0%)	14 (24.6%)	0.002
Waist circumference (cm)	82.9 ± 9.7	91.0 ± 10.4	0.001
BMI (kg/m^2^)	23.8 ± 3.5	26.7 ± 3.5	0.001
SBP (mmHg)	114.4 ± 12.6	127.2 ± 16.7	<0.001
DBP (mmHg)	74.5 ± 7.0	82.1 ± 11.2	0.001
PR (/min)	69.6 ± 11.1	76.1 ± 11.8	0.015
Creatinine (mg/dl)	0.8 ± 0.2	0.8 ± 0.2	0.198
ALT (u/l)	26.0 ± 16.8	38.1 ± 25.3	0.06
Glucose (mg/dl)	89.7 ± 10.5	126.2 ± 31.8	<0.001
HbA1c (%)	5.6 ± 0.4	6.7 ± 0.7	<0.001
Total cholesterol (mg/dl)	186.9 ± 37.1	181.8 ± 41.2	0.64
Triglyceride (mg/dl)	113.9 ± 100.3	132.0 ± 63.9	0.38
LDL-c (mg/dl)	105.4 ± 30.9	112.0 ± 35.1	0.54
HDL-c (mg/dl)	61.1 ± 17.3	43.8 ± 14.6	0.001
Aspirin use	2 (6.7%)	4 (7.0%)	1.000
Statin use	7 (8%)	23 (26.4%)	0.112
ACEI/ARB use	4 (13.3%)	20 (35.1%)	0.043
CCB use	2 (6.7%)	11 (12.6%)	0.204
Oral hypoglycemic agent use	0 (0.0%)	46 (80.7%)	<0.001
TNF-α (pg/ml)	4.1 ± 1.9	4.5 ± 2.9	0.5
IL-6 (pg/ml)	1.6 ± 0.7	2.6 ± 1.6	0.003
hs-CRP (μg/ml)	1.2 ± 1.5	2.4 ± 2.4	0.016
Adiponectin (μg/ml)	8.2 ± 5.7	5.3 ± 3.9	0.007
Resistin (μg/ml)	10.1 ± 6.0	11.0 ± 6.1	0.5

Values are means ± standard deviations or n (%). SBP: systolic blood pressure; DBP: diastolic blood pressure; PR: pulse rate; ALT: alanine aminotransferase; HbA1c: hemoglobin A1c; ACEI/ARB: angiotensin-converting enzyme inhibitor/angiotensin receptor blocker; CCB: calcium channel blocker; TNF-α: tumor necrosis factor-alpha; IL-6: interleukin 6; hs-CRP: high-sensitivity C-reactive protein.

### Increased total and serine protease activities in patients with diabetes

**Figure 1:**
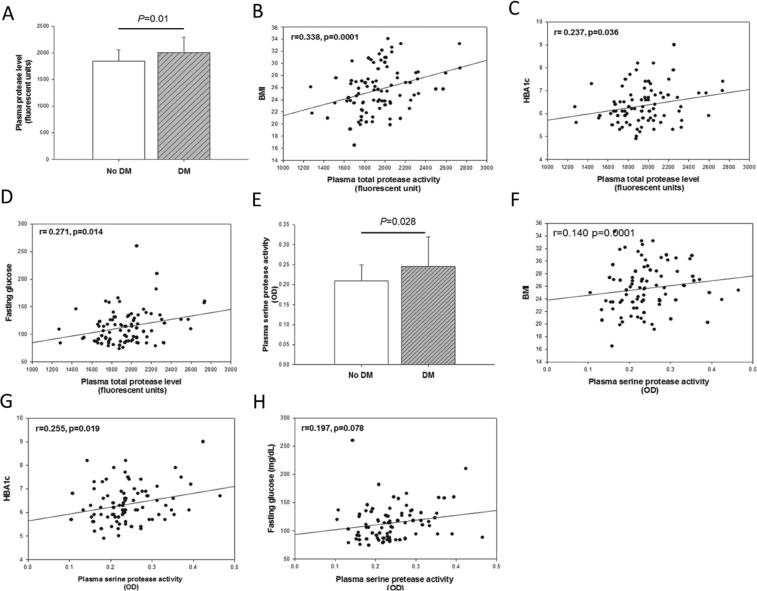
Total and serine protease activity levels were increased in patients with type 2 diabetes mellitus. Total protease activity in plasma (**A**; diabetes, *n* = 57; no diabetes, *n* = 30) was correlated incrementally with hemoglobin A1c (HBA1c) level (**B**), fasting glucose level (**C**), and BMI (**D**). Serine protease activity in plasma (**E**) was correlated incrementally with HBA1c level (**F**), fasting glucose level (**G**), and BMI (**H**).

As shown in Fig. 1A, total plasma protease activity was significantly higher in diabetic patients than in those without diabetes (non-DM vs. DM, 1848 vs. 1999 RFU; p = 0.010). Total plasma protease activity was positively correlated with BMI (r = 0.338, p < 0.001), HBA1c (r = 0.237, p = 0.036), and fasting glucose level (r = 0.271, p = 0.014; Fig. 1B–D). However, there was no significant difference in MMP-9 and MMP-13 levels between groups (Supplementary Fig. 1A).

Additionally, plasma serine protease activity levels were significantly enhanced in patients with diabetes than in those without diabetes (non-DM vs. DM, 0.2092 vs. 0.2457 OD, p = 0.028; Fig. 1E). Plasma serine protease activity was also positively correlated with BMI (r = 0.14, p = 0.0001), HBA1c level (r = 0.255, p = 0.019; Fig. 1F–H), homeostasis model assessment of insulin resistance (HOMA-IR) (r = 0.256, p = 0.017), insulin level (r = 0.213, p = 0.048), and negatively with adiponectin concentration (r = −0.264, p = 0.014). Additionally, serine protease activity was correlated positively with tumor necrosis factor-α (TNF-α) (r = 0.274, p = 0.011), but not with hs-CRP (r = 0.209, p = 0.056), and IL-6 levels (r = 0.017, p = 0.874; Supplementary Fig. 1B–D). However, total protease activity showed no positive correlation with any of the above parameters (Supplementary Fig. 2A–F). Together, these findings indicate that serine protease activity was associated more significantly than total protease activity with type 2 diabetes mellitus.

### LDLR^−/−^ mice fed an HFD developed insulin resistance and type 2 diabetes

**Figure 2:**
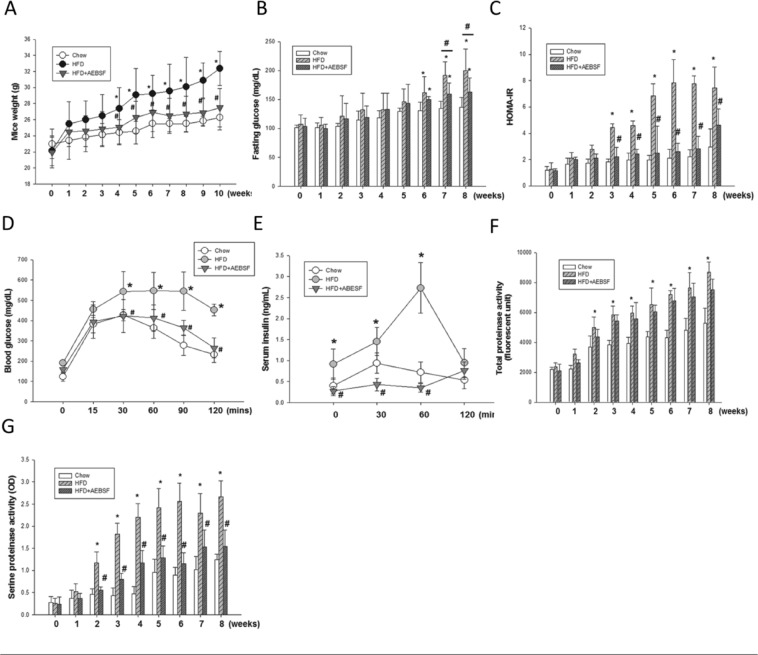
4-(2-Aminoethyl) benzenesulfonyl fluoride hydrochloride (AEBSF) attenuated high-fat diet (HFD)-induced obesity and insulin resistance in male LDLR^−/−^ mice. (**A**) HFD-induced weight gain (*n* = 6–9/group). (**B**) Plasma concentrations of fasting glucose (**B**; *n* = 6−12/group) and Homeostatic Model Assessment of Insulin Resistance (HOMA-IR; **C**; *n* = 6−9/group) in mice receiving HFD and HFD + AEBSF treatments. Plasma concentrations of glucose (**D**) and insulin (**E**) in an intraperitoneal glucose tolerance test for mice receiving the chow, HFD, and HFD + AEBSF treatments (*n* = 6/group). Total (**F**) and serine (**G**) protease activities in mice receiving the chow, HFD, and HFD + AEBSF treatments (*n* = 6/group) *P < 0.05, chow vs. HFD; ^♯^*p* < 0.05, HFD vs. HFD + AEBSF. Values are means ± standard deviations.

Compared with mice fed the normal chow diet, HFD-fed mice showed significant body weight increase at 4 weeks (25.5 ± 1.0 vs. 27.6 ± 1.7 g, p < 0.05; Fig. 2A) and higher fasting glucose level at 6 weeks (130.6 ± 15.0 vs. 161.8 ± 28.1 mg/dl, p < 0.05; Fig. 2B). Besides, HOMA-IR was significantly higher in HFD-fed mice after 3 weeks (4.4 ± 0.3 vs. 1.8 ± 0.2, p < 0.05; Fig. 2C). Consistent with these findings, HFD-fed mice had higher glucose (542.6 ± 99.3 vs. 427.3 ± 27.9 mg/dl, p < 0.05; Fig. 2D) and serum insulin (1.4 ± 0.3 vs. 0.9 ± 0.2 ng/ml, p < 0.05; Fig. 2E) levels than did standard chow-fed mice at 30 min after the IPGTT glucose challenge. Compared with chow-fed mice, HFD-fed mice had significantly higher plasma total protease (3703.6 ± 723.0 vs. 4994.4 ± 711.2 RFU, p < 0.05; Fig. 2F) and serine protease (0.4 ± 0.1 vs. 1.1 ± 0.2 OD, p < 0.05; Fig. 2G) activity levels from week 2. Circulating adiponectin levels were decreased by 31%, and leptin markedly increased in HFD-fed mice compared with chow-fed mice at week 10 (Table 2). These data suggest that the HFD induced upregulation of plasma total protease and serine protease activities followed by insulin resistance, weight gain, blood glucose elevation and finally alteration of adipokines in LDLR^−/−^ mice.

**Table 2:** Metabolic parameters at 10 weeks of dietary control in LDLR^−/−^ mice.

	Chow (*n* = 7)	HFD (*n* = 9)	HFD + AEBSF (*n* = 9)
Glucose (mg/dl)	105.4 ± 15.5	205.3 ± 24.2^*^	155.5 ± 18.6^#&^
Cholesterol (mg/dl)	255.7 ± 42.5	974.7 ± 95.2^*^	519 ± 65.4^#&^
HDL-c (mg/dl)	60.1 ± 11.1	285.8 ± 15.3^*^	239.5 ± 40.8^#&^
LDL-c (mg/dl)	91 ± 17.9	537.3 ± 43.3^*^	429 ± 90.4^#&^
Triglycerides (mg/dl)	109.3 ± 8.5	338.6 ± 48.2^*^	230 ± 16.6^#&^
TNF-α (pg/ml)	1.4 ± 0.22	3.2 ± 0.4^*^	2.4 ± 0.2^#&^
IL-6 (pg/ml)	5.8 ± 2.0	19.4 ± 6.2^*^	14.8 ± 5.7^#&^
Leptin (μg/ml)	0.94 ± 0.27	6.60 ± 2.41^*^	2.71 ± 0.68^#&^
Adiponectin (μg/ml)	38.12 ± 8.3	26.19 ± 4.82^*^	36.3 ± 5.27^#^
Resistin (μg/ml)	14.59 ± 4.4	25.56 ± 7.57^*^	22.43 ± 8.16^&^
BUN (mg/dl)	25 ± 1	34 ± 6.7^*^	30.2 ± 3.5^&^
Creatinine (mg/dl)	0.12 ± 0.01	0.2 ± 0.08^*^	0.22 ± 0.06^&^
Total bilirubin (mg/dl)	0.26 ± 0.07	0.12 ± 0.1^*^	0.36 ± 0.1^#&^
ALP (u/l)	68.2 ± 15	62.9 ± 15	83 ± 19.3^#^

Values are means ± standard deviations. *p < 0.05, chow vs. HFD; #p < 0.05, HFD vs. HFD + AEBSF; ^&^p < 0.05, Chow vs. HFD + AEBSF. HFD: high-fat diet; AEBSF: 4-(2-aminoethyl) benzenesulfonyl fluoride hydrochloride; TNF-α: tumor necrosis factor-alpha; IL-6: interleukin 6; BUN: blood urea nitrogen; ALP: alkaline phosphatase.

### Effects of serine protease inhibitor on fasting glucose level and insulin resistance in LDLR^−/−^ mice fed an HFD

Compared with the chow diet, the HFD significantly increased fasting glucose, total cholesterol, HDL-c, LDL-c, and triglyceride levels, and decreased the total bilirubin level, in LDLR^−/−^ mice; AEBSF attenuated these effects (all p < 0.05; Table 2). AEBSF treatment significantly increased total bilirubin levels compared to HFD or chow control (Table 2). AEBSF treatment also elevated alkaline phosphatase levels compared to HFD group (Table 2). AEBSF treatment significantly lessened the TNF-α level and upregulated the circulating adiponectin concentration (both p < 0.05; Table 2). As expected, AEBSF inhibited plasma serine protease activity at 2 weeks (1.1 ± 0.2 vs. 0.5 ± 0.07 OD, p < 0.05; Fig. 2D), but did not suppress total protease activity (Fig. 2E).

AEBSF treatment resulted in significant reductions in HFD-induced body weight gain (Fig. 2A), fasting blood glucose level (Fig. 2B), and HOMA-IR (Fig. 2C). At 10 weeks, glucose and insulin levels were significantly improved in mice treated with AEBSF compared with vehicle-treated mice (30 min after i.p. glucose injection: glucose, 423.0 ± 81.4 vs. 542.6 ± 99.3 mg/dl; insulin, 0.4 ± 0.1 vs. 1.4 ± 0.3 ng/ml; both p < 0.05; Fig. 2F–G). These results suggest that AEBSF attenuated HFD-induced insulin resistance in LDLR^−/−^ mice.

### Serine protease inhibitor improved the decreased insulin signaling in visceral fat tissue

**Figure 3:**
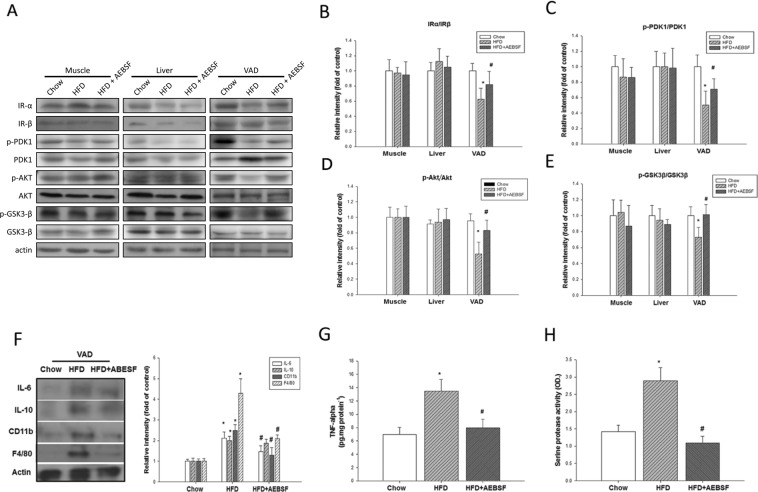
4-(2-Aminoethyl) benzenesulfonyl fluoride hydrochloride (AEBSF) recovered high-fat diet (HFD)-impaired insulin signaling and inflammation in visceral adipose (VAD) tissue of LDLR^−/−^ mice. (**A**) The peripheral-tissue insulin signaling pathway [insulin receptor, pyruvate dehydrogenase kinase isozyme 1 (PDK1), Akt, glycogen synthase kinase 3β (GSK3β)] was analyzed by western immunoblots. (**B**) Quantification of insulin receptor-α and -β expression, measured by imageQuant. Amounts of phosphorylated and total PDK1 (**C**), Akt (**D**), and GSK3β (**E**). (**F**) VAD tissue inflammatory cytokines and macrophage markers [interleukin (IL)-6, IL-10, CD11b, F4/80] were analyzed by western immunoblots, with protein levels quantified by imageQuant. (**G**) Tumor necrosis factor-α (TNF-α) expression in VAD tissue was analyzed by ELISA. (**H**) Serine protease activity in VAD tissue of mice receiving the chow, HFD, and HFD + AEBSF treatments (*n* = 6/group). *P < 0.05, chow vs. HFD; ♯*p* < 0.05, HFD vs. HFD + AEBSF. Values are means ± standard deviations. Full-length blots were presented in Supplementary Figs. 3 and 4.

HFD-fed mice showed a reduced ratio of protein levels of insulin receptors α and β in visceral adipose (VAD) tissue, but there was no significant effect in muscle or liver tissue (Fig. 3A–E). AEBSF treatment significantly reversed this effect in VAD tissue compared with HFD-fed mice (0.8 ± 0.2 vs. 0.6 ± 0.2, p < 0.05; Fig. 3A–E). Protein levels indicated that phosphorylation of pyruvate dehydrogenase kinase isozyme 1 (PDK1), Akt, and glycogen synthase kinase 3β (GSK3β) was downregulated in VAD tissue, but not in muscle or liver tissue. Unlike Akt that has to be phosphorylated before activation, GSK3β is constitutively active in resting cells, requiring phosphorylation by kinases such as AKT to inactivate it. AEBSF treatment resulted in a modest recovery of downregulation of PDK1, Akt, and upregulation of GSK3β in VAD tissue (all p < 0.05). Thus, insulin signaling was downregulated in VAD tissue in LDLR^−/−^ mice fed a HFD, and AEBSF induced partial recovery from this suppression effect.

The HFD promoted serine protease activation; IL-6, IL-10, and TNF-α proteins expression; and upregulation of the macrophage-related markers CD11b and F4/80 in VAD tissue (Fig. 3F–H). AEBSF significantly downregulated these activities, except for IL-10 expression. Together, the HFD induced serine protease activity and triggered inflammatory cytokines secretion, and AEBSF attenuated these effects.

## Discussion

The study results suggest that circulating serine protease activity is significantly elevated and associated with HBA1c, HOMA-IR, TNF-α, and adiponectin levels in patients with type 2 diabetes. Animal experiments in LDLR^−/−^ mice confirmed these findings that administration of HFD produced progressive hyperglycemia and insulin resistance associated with upregulation of serine protease activity in plasma and VAD tissue. By showing a reduced ratio of protein levels of insulin receptors-α and β in VAD, we suggest that HFD will produce excessive proteolytic cleavage of the extracellular domain of insulin receptors in VAD (not in liver nor muscle tissue), thereby further advancing the knowledge in this field. It is worth noting that the treatment of the selective serine protease inhibitor AEBSF significantly attenuated the diabetogenic effects of HFD through the suppression of systemic inflammation, enhancement of adiponectin concentration, and improvement of insulin sensitivity in this animal model.

The pathogenesis of obesity-induced diabetes is multifactorial and complex. α1-Antitrypsin is known to play a pivotal role in blocking the early stage of proinflammatory cytokine production in mice with HFD-induced obesity^5^. Serine protease activation is likely involved in the inflammation of HFD-induced obesity. These studies clarified serine protease expression in subjects with diabetes mellitus and its action in diet-induced obesity. We showed that patients with diabetes have greater total and serine protease activities, in association with higher BMI and systemic inflammation. Moreover, plasma serine protease activity was positively correlated with BMI, HBA1c, fasting glucose levels, HOMA-IR, TNF-α, and also negatively with adiponectin concentration. However, the total protease activity showed no significant correlation with any of the above parameters. These findings provide novel evidence that serine protease activity was associated more significantly than total protease activity in patients with type 2 diabetes mellitus.

Obesity has been established as an important factor in the etiology of insulin resistance and type 2 diabetes^13^. Tissue factor protease-activated receptor 2 signaling was shown to promote diet-induced obesity and insulin resistance^3^. In the present study, glucose level and HOMA-IR were significantly elevated in HFD-fed than in chow-fed LDLR^−/−^ mice. These results suggest that the glucose challenge mediated insulin secretion in the circulation, but the increased insulin had only a minor effect on the promotion of glucose uptake by peripheral tissue. These phenomena were confirmed by the IPGTT, in which the glucose challenge increased blood glucose and insulin levels and reduced glucose clearance in the circulation. Besides, HFD-induced increases in systemic inflammation and serine protease activity were also observed in LDLR^−/−^ mice. Inhibition of serine protease by AEBSF diminished the effects of the HFD on HOMA-IR, fasting glucose level, and blood glucose and insulin levels in the IPGTT, confirming that inhibition of serine protease reduced HFD-induced insulin resistance in LDLR^−/−^ mice. Our data are in line with previous reports that reduced α1-antitrypsin levels were correlated negatively with BMI in obese humans^5^, and HFD-induced insulin resistance is attenuated by adipose tissue-derived serpins^9^.

Proteases derived from diverse sources are important mediators of HFD-induced obesity and diabetes. An HFD appears to cause protease and antiprotease imbalances in circulation^5^. Our study showed that inhibition of serine protease recovered HFD-related impaired insulin signaling in VAD tissue in LDLR^−/−^ mice, which suggests specific suppression of serine protease activity may prevent the destruction of the extracellular domain of insulin receptor-α, and protects insulin receptors. These findings support previous reports about the soluble insulin-receptor ectodomain elevated in human obesity and type 2 diabetes mellitus^10,11^. Additionally, we extended previous findings showing that HFD produced excessive proteolytic cleavage of extracellular domain of insulin receptors in VAD not in liver nor muscle tissues. The excessive cleavage resulted in decreased detection of insulin receptor-α and impaired insulin signaling cascade in VAD in this study. Little is known about insulin receptor-α in animal study. Recently, it was reported that hyperinsulinemia would promote proteolytic cleavage of insulin receptor-α in rat hepatocyte culture^14^. They found increased proteolytic cleavage of insulin receptor in culture medium with higher insulin concentration^14^. HFD induced more complex changes in mice than simply hyperinsulinemic condition. Elevated serine protease activity in VAD, not liver, seems to play an important role in HFD-induced obesity in our study.

A variety of immune cells have been shown to participate in the complex intracellular communication network that organizes the chronic inflammatory response to obesity^4^. TNF-α and IL-6 are considered to be contributors to insulin resistance and diabetes mellitus development^15,16^. In this study, AEBSF attenuated increases in TNF-α and IL-6 levels in HFD-fed mice. Serine protease could be considered to be an active participant in obesity-induced inflammation and insulin resistance. These results are consistent with those of previous studies showing that polymorphonuclear neutrophil-derived serine proteases hydrolyze peptide bonds of TNF-α precursors and produce mature cytokine molecules^17^. Targeting inflammatory pathways could possibly be a component of the therapeutic strategies to prevent and control insulin resistance and diabetes.

Furthermore, adiponectin and leptin, an adipokine produced exclusively by adipocytes, are known to have critical effects on body weight regulation^18^. In this study, circulating adiponectin levels were significantly lower in participants with diabetes and HFD-fed LDLR^−/−^ mice compared with controls. Selective inhibition of serine protease attenuated HFD-suppressed adiponectin levels. These findings are in agreement with the previous demonstration of improved insulin sensitivity in vaspin-suppressed obese mice fed high-fat, high-sucrose chow, as reflected by leptin and TNF-α downregulation and adiponectin upregulation^19^. Lower circulating adiponectin levels have been observed in patients with obesity and type 2 diabetes compared with lean subjects, and have been associated with insulin sensitivity, lipid and glucose metabolism, and inflammation^20,21^. AEBSF attenuated HFD-induced circulating IL-6 accumulation and recovered HFD-suppressed circulating adiponectin production. It also attenuated HFD-increased IL-6, CD11b, F4/80, and TNF-α expression and serine protease activity in VAD tissue. These data suggest that serine protease activity inhibition reduces adipose tissue inflammation, improving insulin sensitivity, in HFD-fed LDLR^−/−^ mice. Endogenous serine protease inhibitor may exert insulin-sensitizing effects on white adipose tissue in various states of obesity^19^.

Previous studies have shown that administration of an endogenous serine protease inhibitor is correlated negatively with BMI and overcomes insulin resistance in obese mice^5,19^. However, details of the underlying mechanisms remained largely unknown. To clarify whether and how a serine protease inhibitor could modify insulin sensitivity, three kinases in the insulin signaling cascade (PDK1, Akt, and GSK3β) were examined in liver, muscle, and VAD tissues in HFD-fed LDLR^−/−^ mice. Glucose and insulin levels were higher in HFD-fed mice after the IPGTT glucose challenge, and were reduced by AEBSF. In addition, AEBSF recovered the baseline phosphorylation status of all three kinases in VAD tissue, but not in liver or muscle tissue, in HFD-fed mice, indicating the selective tissue-specific improvement of insulin sensitivity. AEBSF mediated the recovery of insulin receptor-α/β, phosphorylation of PDK1, Akt, and GSK3β protein levels, contributing, at least partially, to resistance to HFD-induced insulin signaling loss in LDLR^−/−^ mice. Our findings provide initial evidence suggesting that insulin receptors in VAD tissue are the main targets of serine protease inhibition, which reduces systemic inflammation and insulin resistance in experimental diabetes. VAD is known to play a critical pathophysiological role in clinical metabolic syndrome and type 2 diabetes. Interestingly, a recent study reported that the amount of biologically insulin receptor active is regulated by the cleavage of its ectodomain, by the β-site amyloid precursor protein cleaving enzyme 1 (BACE1), in a glucose concentration-dependent manner^22^. They demonstrated that BACE1 (an aspartyl protease, not serine protease) regulated the cleavage of insulin receptor and insulin signaling mainly in *db/db* mouse livers^22^. In contrast, we confirmed upregulation of serine protease in VAD (not liver or muscle) play some role in HFD-induced insulin resistance in LDLR^−/−^ mice in concordance with the previous studies^5,19^. We speculated that different pathophysiological mechanisms may exist in different diabetic models (HFD-induced diabetes vs. *db/db* mice). Further studies are warranted to validate this speculation.

This study has several limitations. First, many proteases are active in blood^23^. Serine protease was shown to exert a crucial role in HFD-induced diabetes development, but interaction by other proteases in this study cannot be ruled out. Second, we investigated total serine protease activities in blood and VAD but not specific protease such as vaspin. The identity of the serine protease involved in obesity-associated diabetes development remains unclear; future studies should use activity-based probes to identify potential candidates. Third, the influence of serine hydrolase, which has a similar active site as serine protease^23^, was not investigated in the present study; its function may also be blocked by AEBSF. That is, the attenuation of serine hydrolase may have contributed to the improvement in lipid metabolism after AEBSF treatment in LDLR^−/−^ mice^24^. Also, it would be important to understand if there is a combined action to generate the improvement in lipid metabolism after AEBSF treatment. In addition, we explored the insulin signaling pathway by investigating tissue insulin receptor-α and three kinases in the signaling cascade, PDK1, Akt, and GSK3β. The other important parameters (insulin receptor substrate 1 and glucose transporter type 4 in signaling cascade and plasma soluble insulin receptor-α) may provide further information about the roles of insulin receptor-α/β in insulin receptor function and signalling. The study is still preliminary and the nature of human and mouse plasma and VAD proteases need to be established. Furthermore, the potential side effect of AEBSF is unclear. In this study, we found significantly elevated levels of total bilirubin in AEBSF treatment group compared to chow diet and HFD group.

**Figure 4:**
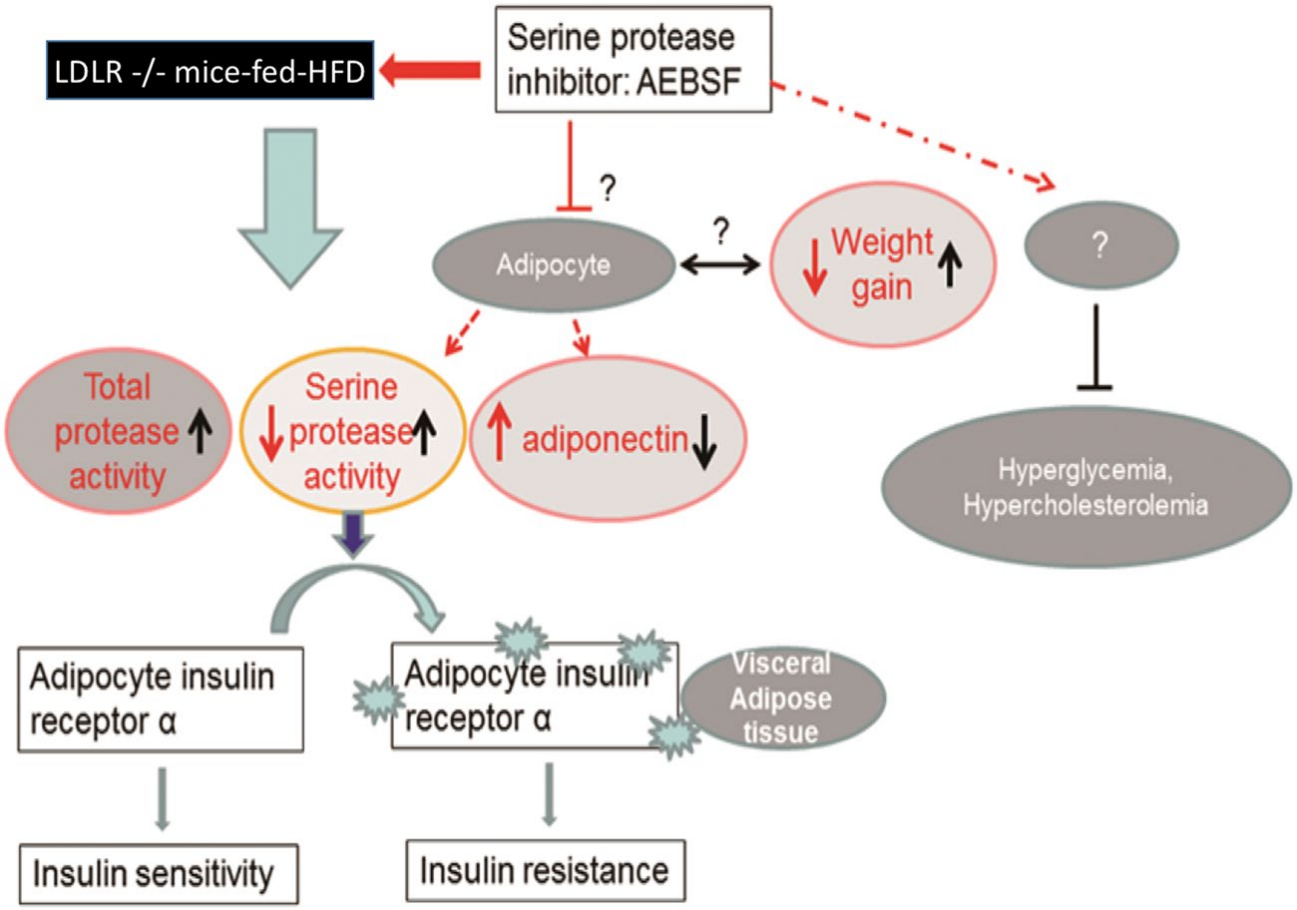
Schematic overview of the contributions of serine protease to insulin resistance and obesity in high-fat diet (HFD)-fed LDLR^−/−^ mice. These mice showed increased plasma total and serine protease activities, weight gain, and attenuated accumulation of insulin receptor-α in visceral fat tissue. Serine protease destroyed insulin receptor-α, contributing to insulin resistance. 4-(2-Aminoethyl) benzenesulfonyl fluoride hydrochloride (AEBSF) reversed insulin resistance and weight gain. However, the detailed mechanism of the weight gain reversal, adiponectin revision, and cholesterol and blood glucose lowering effects remains unclear.

In conclusion, serine protease activity is increased in clinical and experimental diabetes, which may be critical for type 2 diabetes development. As depicted in Fig. 4, the specific serine protease inhibitor AEBSF attenuated systemic inflammation, obesity, and insulin resistance in diabetic mice, probably by modulating insulin receptor and cytokine expression in VAD tissue. Our findings support the potential role of serine proteinase as the therapeutic target for clinical prevention of type 2 diabetes. Further clinical studies are required to verify this concept.

## Methods

### Human study: population, design, and measurements

We conducted a single-center, cross-sectional study with 87 subjects from Taipei Veterans General Hospital, Taipei, Taiwan. Study subjects were divided into two groups: type 2 diabetes mellitus and no diabetes. Diabetes mellitus was diagnosed by anti-hyperglycemic agent use or American Diabetes Association criteria^25^. All subjects apart from those reporting the prior diagnosis of diabetes underwent a standard 75-g oral glucose tolerance test, which included the measurement of fasting glucose. Subjects were excluded using the following criteria: (1) age < 18 years or >80 years, (2) receipt of insulin or insulin analog, (3) renal and/or liver function impairment, and (4) HbA1c > 8.0% (64 mmol/mol). The study protocol was approved by Institutional Review Board of Taipei Veterans General Hospital, Taipei, Taiwan (VGHIRB no. 201001004IC). All participants provided written informed consent before entering the study. In addition, all methods were performed in accordance with relevant guidelines and regulation.

Blood samples were collected after a ≥12-h fast and examined using biochemical tests to determine creatinine, alanine aminotransferase (ALT), glucose, HbA1c, and lipid profiles. To examine the role of circulating proteases in type 2 diabetes, plasma total and serine protease activities were determined. Protease-related markers, such as MMP-9 and MMP-13, were analyzed by Quantikine human ELISA kits (R&D Systems, Minneapolis, MN).

### HFD-induced diabetes mouse model

We used a murine model to clarify the role of serine protease in obesity and dysglycemia development. All experimental procedures and protocols involving animals were approved by the institutional animal care committee of National Yang-Ming University, Taipei, Taiwan [IACUC approval no. IACUC-1030306 (YM)], and were carried out in accordance with the Guidelines for the Care and Use of Laboratory Animals. Thirty 8-week-old male LDLR^−/−^ mice (C57BL/6 J background) were obtained from The Jackson Laboratory (Sacramento, CA, USA), and housed under standard 12/12-h light/dark conditions. They were divided randomly into a chow diet control group, a HFD group (positive control), and a HFD + AEBSF group. The chow diet (Picolab Rodent Diet 20; 4 kcal/g, 2% cholesterol, 5.7% fat) and HFD (Teklad diet TD 88137; 4.5 kcal/g, 0.2% cholesterol, 21.2% fat; Harlan Tackle Co.) were administered for 10 weeks. An HFD is known to trigger the development of obesity and type 2 diabetes mellitus in LDLR^−/−^ mice^12^. Mice in the HFD + AEBSF group received AEBSF (5 mg/kg/day; once a day, 5 days/week, intraperitoneal injection), and those in the HFD control group received PBS (100 μl/day, 5 days/week, intraperitoneal injection), for 10 weeks. AEBSF (Sigma, St. Louis, MO, USA), which inhibits serine protease activity by irreversible covalent bonding to the enzyme, was administered to clarify whether the pharmacological inhibition of serine protease could reverse obesity and improve insulin sensitivity in HFD-fed mice.

### Measurements

Food intake, body weight, and blood glucose and insulin levels were recorded weekly. Mice were fasted overnight, and blood samples were collected from the facial vein. The intraperitoneal glucose tolerance test (IPGTT) was administered at 9 weeks, as described previously^26^. Briefly, after overnight fasting, each mouse was given an intraperitoneal injection of 1 g glucose kg^−1^ body weight using 50% glucose in 0.9% NaCl. Blood glucose concentrations were measured from a nicked tail vein at 0 (baseline), 30, 60, and 120 min after glucose injection using a blood glucose meter. Blood collected at these time points was isolated for the detection of circulating insulin levels.

After 10 weeks of treatment, mice were fasted overnight and sacrificed by exsanguination under adequate anesthesia [ketamine-HCl (100 mg/kg) and xylazine (20 mg/kg), intraperitoneal injection). The thoracic cavity was opened, a blood sample was collected, and the liver, VAD, and muscle tissues were isolated, frozen quickly in liquid nitrogen, and stored at −80 °C for later analysis. To explore the molecular mechanism involved in the activation of serine protease-regulated metabolic functions, these tissues were used to quantify insulin signaling. Mouse serum was used to quantify lipid, adipokine, and cytokine levels.

Fasting serum insulin, glucose, total cholesterol, triglyceride, LDL-c, HDL-c, creatinine, blood urea nitrogen (BUN), aspartate transaminase, and ALT levels were measured. The HOMA-IR was used to calculate insulin resistance. Glucose was assessed with a glucose meter (Optium Xceed™ diabetes monitoring system; Vic., Australia). Serum fasting insulin, leptin, adiponectin (an insulin-sensitizing adipokine), resistin, TNF-α, and hs-CRP levels were determined by ELISA (R&D Systems, Minneapolis, MN).

### Reagents

Mouse TNF-α and fluorescent protease assay kits were obtained from Life Technologies (Grand Island, NY, USA). Antibodies against mouse insulin receptor-α (ab-78424), mouse insulin receptor-β (ab-80527), cluster of differentiation molecule 11b (CD11b) (ab-75476), and F4/80 (ab-6640) were obtained from Abcam Inc. (Cambridge, UK). Antibodies against mouse PDK1 (#3062), phospo-PDK1 (#3438), Akt (#9272), phospho-Akt (#9271), GSK3β (#9315), and phospho-GSK3β (#9336) were obtained from Cell Signaling Technology (Danvers, MA, USA). Antibodies against mouse interleukin (IL)-6 (MAB-406) and IL-10 (AF-519) and all proteins were detected using horseradish peroxidase (HRP)-conjugated secondary antibodies (R&D Systems, Minneapolis, MN). Antibodies against mouse actin (sc-47778) and all proteins were detected using HRP-conjugated secondary antibodies (Santa Cruz Biotechnology, Santa Cruz, CA, USA). Unless otherwise specified, all other chemicals and reagents were obtained from Sigma-Aldrich (St. Louis, MO, USA).

### Glucose homeostasis

HOMA-IR was calculated as [fasting insulin (pmol/l) × fasting glucose (mmol/l)]/135^27^. The early secretory response of insulin to an oral glucose load (Δinsulin 30 − 0/Δglucose 30 − 0) was calculated as [Δ insulin (30 min − 0 min)]/[Δ glucose (30 min − 0 min)]. The incremental (above the baseline value) areas under the curve for glucose and insulin were calculated using GraphPad 5.0.

### Western blot analysis

Skeletal muscle, liver, and VAD tissue lysates were prepared by dissection and homogenized in buffer (25 mM HEPES, pH 7.4; 1% Nonidet P-40; 137 mM NaCl; 1 mM phenylmethylsulfonyl fluoride, 10 μg/ml aprotinin, 1 μg/ml pepstatin, 5 μg/ml leupeptin) using a PRO 200 homogenizer (PROScientific, Oxford, CT, USA). The samples were centrifuged at 14,000 × g for 20 min at 4 °C, and supernatant protein concentrations were determined using a protein assay kit (Bio-Rad Laboratories, Inc., Hercules, CA, USA). Supernatants (50 μg) were resolved by SDS-PAGE and subjected to immunoblotting. Protein abundance was detected with antibodies against insulin receptor-α, insulin receptor-β, Akt1, phospho-Akt1, PDK1, phospho-PDK1, GSK3β, phospho-GSK3β, and β-actin. All proteins were detected with HRP-conjugated secondary antibodies using Chemiluminescence Reagent Plus (PerkinElmer Life Science, Boston, MA, USA), and quantified with a densitometer. All proteins were normalized by β-actin, and specific protein phosphorylation was normalized by corresponding proteins.

### Casein protease activity assay

To assess the ability of plasma proteases to digest large globular proteins, we utilized the Enzchek protease assay kit (Enzchek BODIPY, casein derivative, catalogue no. E-6638; Molecular Probes, Carlsbad, CA, USA; cleaved by metallo-, serine, acid, and sulfhydryl proteases), which consists of casein internally quenched with Texas FITC fluorophores (Ex/Em: 505/513 nm) reconstituted in a digestion buffer. Plasma samples were tested simultaneously for overall protease activity. Protease activity levels were determined from fluorescent intensity after peptide cleavage following 18 h incubation at 37 °C (SpectraMax Gemini XS; Molecular Devices, Sunnyvale, CA, USA).

### Serine protease activity assay

Human and mouse plasma serine protease activities were measured with suc-AAPF-pNA, a highly specific synthetic substrate for serine protease, according to methods described previously^28^. Mouse plasma and VAD tissue were thawed by addition to 1 ml tissue lysis buffer (2 mM Tris, pH 7.2; 100 μM NaN_3_; 50 mM NaCl; 0.1% IGEPAL). Tissue was homogenized and centrifuged at 12,000 × g. Supernatants were then centrifuged in 0.22-μm SPIN-X filter tubes. For the protease activity assay, suc-AAPF-pNA was dissolved in dimethyl sulfoxide, and 1 μl 40-mM solution was added to a 100-μl volume of 10 μl mouse plasma or 0.5 mg/ml lysate protein in tissue lysis buffer. Cleavage of suc-AAPF-pNA was followed by absorbance at 405 nm for 18 h incubation.

### Statistical analysis

Continuous variables were expressed as means ± standard deviations, and categorical variables were presented as frequencies and percentages. Differences between groups were analyzed using two-tailed Student’s t tests. Subgroup comparisons of categorical variables were performed using the chi-squared test and Fisher exact test. Correlations of plasma total and serine protease activities with variables in the study groups were calculated using Pearson’s product-moment correlation analysis. All tests were two sided and p < 0.05 was considered to be statistically significant. Statistical analyses were performed with SPSS software (version 17; SPSS Inc., Chicago, IL, USA).

## Supplementary information

Supplementary Information .

## Data Availability

The datasets generated during and/or analysed during the current study are available from the corresponding author on reasonable request.
