# Supplementary material for: Inhibition of Serine Protease Activity Protects Against High Fat Diet-Induced Inflammation and Insulin Resistance
Source: Sci Rep. 2020 Feb 3;10:1725. doi: 10.1038/s41598-020-58361-4 (PMC6997356; doi:10.1038/s41598-020-58361-4)
Supplement: Supplementary file 1 — Supplementary Information . [file 41598_2020_58361_MOESM1_ESM.docx]

**Inhibition of Serine Protease Activity Protects Against High Fat Diet-Induced Inflammation and Insulin Resistance**

Chin-Sung Kuo^1^, Jia-Shiong Chen^2^, Liang-Yu Lin^3^, Geert W. Schmid-Schönbein^4^, Shu Chien^5^, *Po-Hsun Huang^6^, *Jaw-Wen Chen^7^, *Shing-Jong Lin^8^

1. **Chin-Sung Kuo**, MD. Division of Endocrinology and Metabolism, Department of Medicine, Taipei Veterans General Hospital and Institute of Clinical Medicine, National Yang-Ming University, Taipei, Taiwan. **E-mail:** cskuo@vghtpe.gov.tw
2. **Jia-Shiong Chen**, PhD. Institute of Clinical Medicine, National Yang-Ming University, Taipei, Taiwan. **E-mail:** jschen@ym.edu.tw
3. **Liang-Yu Lin**, MD. Division of Endocrinology and Metabolism, Department of Medicine, Taipei Veterans General Hospital, Taipei, Taiwan. **E-mail:** tristan074@gmail.com
4. **Geert W. Schmid-Schönbein**, PhD. The Institute of Engineering in Medicine, University of California San Diego La Jolla, California, US. **E-mail:** gwss@ucsd.edu
5. **Shu Chien**, PhD. Departments of Bioengineering, Nanoengineering, Institute of Engineering in Medicine, University of California San Diego La Jolla, California, US. **E-mail:** shuchien@ucsd.edu
6. **Po-Hsun Huang**, MD, PhD. Division of Cardiology, Department of Critical Care Medicine, Taipei Veterans General Hospital and Institute of Clinical Medicine, and Cardiovascular Research Center, National Yang-Ming University, Taipei, Taiwan. **E-mail:** huangbs@vghtpe.gov.tw
7. **Jaw-Wen Chen**, MD. Department of Medical Research and Education, Taipei Veterans General Hospital, Institute and Department of Pharmacology, and Cardiovascular Research Center, National Yang-Ming University, Taipei, Taiwan. **E-mail:** jwchen@vghtpe.gov.tw
8. **Shing-Jong Lin**, MD, PhD. Healthcare and Services Center, Taipei Veterans General Hospital, Institute of Clinical Medicine, and Cardiovascular Research Center, National Yang-Ming University, and Taipei Heart Institute, Taipei Medical University, Taipei, Taiwan. **E-mail:** sjlin@vghtpe.gov.tw

***Corresponding authors:**

* Professor Po-Hsun Huang, MD, PhD

Department of Critical Care Medicine, Taipei Veterans General Hospital

No. 201, Sec. 2, Shih-Pai Road, Taipei, Taiwan

Tel: + 886-2-2875-7434; Fax: + 886-2-2875-7435

**E-mail:** [huangbs@vghtpe.gov.tw](mailto:huangbs@vghtpe.gov.tw)

&

*Professor Jaw-Wen Chen, MD,

Department of Medical Research,

Taipei Veterans General Hospital, Taipei, Taiwan.

112, No. 201, Sec. 2, Shih-Pai Road, Taipei, Taiwan.

**E-mail:** jwchen@vghtpe.gov.tw

&

*Professor Shing-Jong Lin, MD, PhD,

Healthcare and Services Center,

Taipei Veterans General Hospital, Taipei, Taiwan.

112, No. 201, Sec. 2, Shih-Pai Road, Taipei, Taiwan.

**E-mail:** sjlin@vghtpe.gov.tw

**Supplementary figure legends**

**
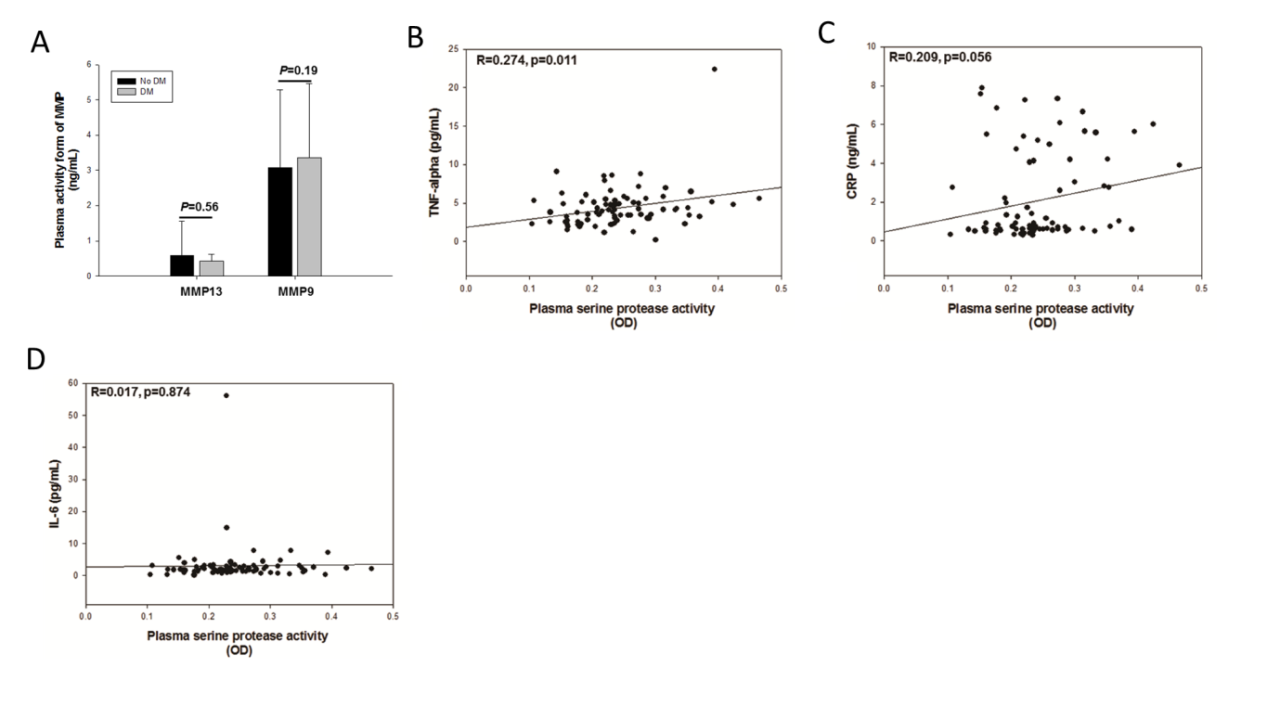
Supplementary Figure 1.** (*A*) Matrix metallopeptidase 9 (MMP 9) and matrix metallopeptidase 13 (MMP 13) activity levels in plasma showed no significant difference between subjects with diabetes (DM, *n* = 57) and without diabetes (no DM, *n* = 30). Plasma serine protease activity was correlated positively with TNF-α (*B*), but not with high-sensitivity C-reactive protein (hs-CRP) (*C*), and interleukin (IL)-6 levels (*D*).


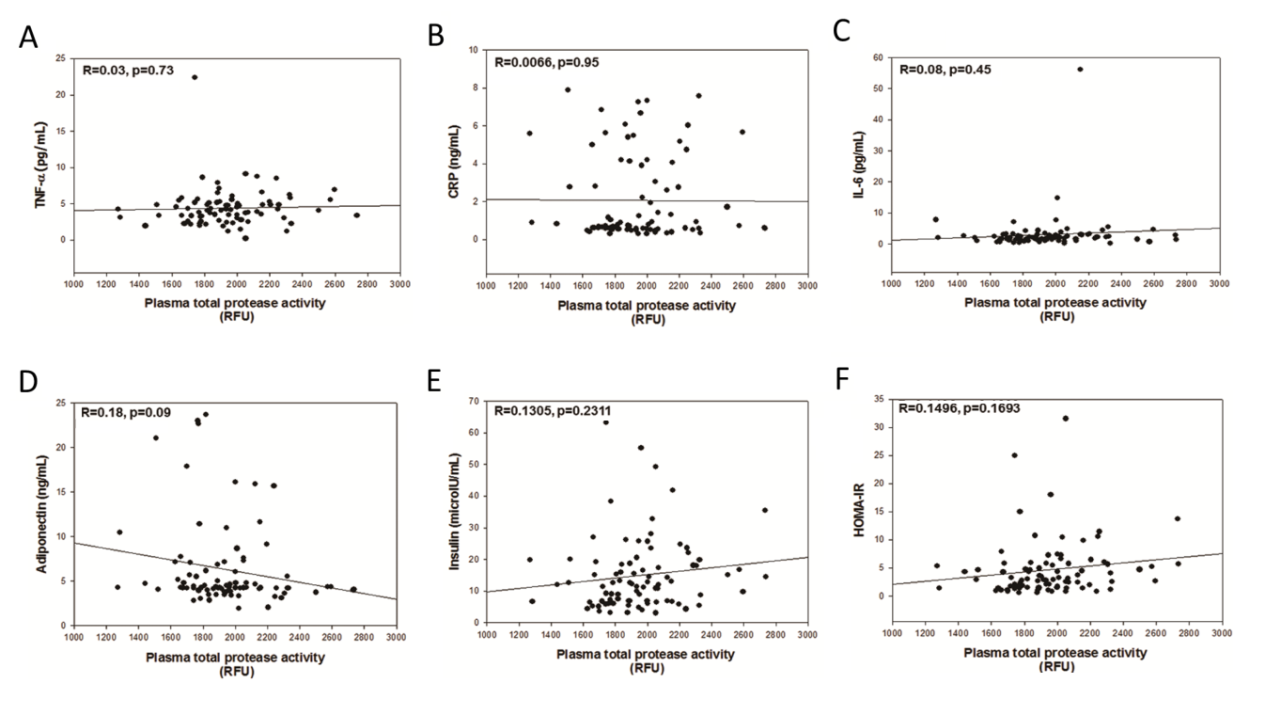


**Supplementary Figure 2.** Plasma total protease activity showed no significant correlation with tumor necrosis factor-α (TNF-α) (*A*), high-sensitivity C-reactive protein (hs-CRP) (*B*), interleukin (IL)-6 (*C*), adiponectin (*D*), insulin (*E*), Homeostatic Model Assessment of Insulin Resistance (HOMA-IR) (*F*).

**Supplementary Figure 3: Full length blots for Fig. 3A
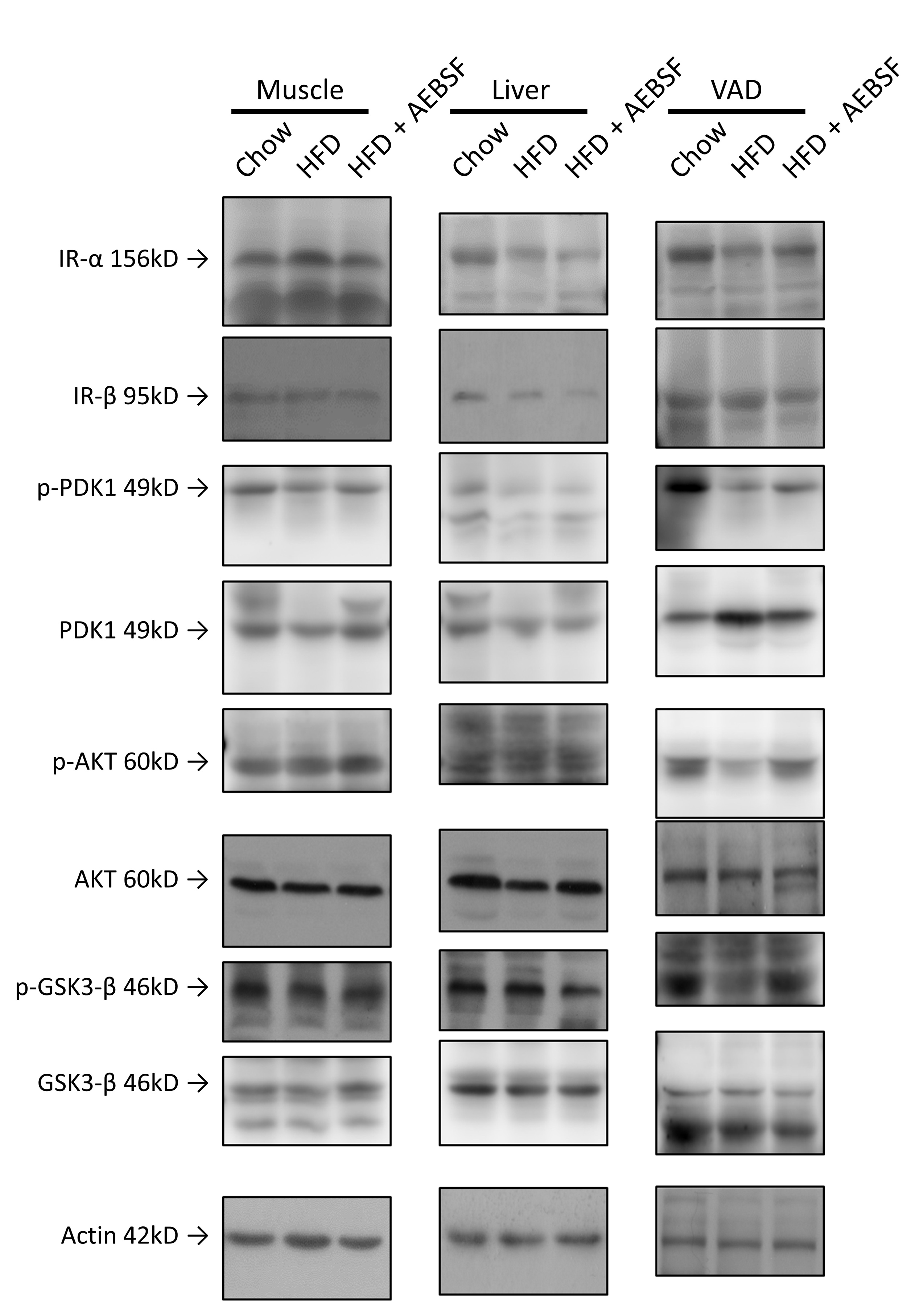
**

**Supplementary Figure 4: Full length blots for Fig. 3F
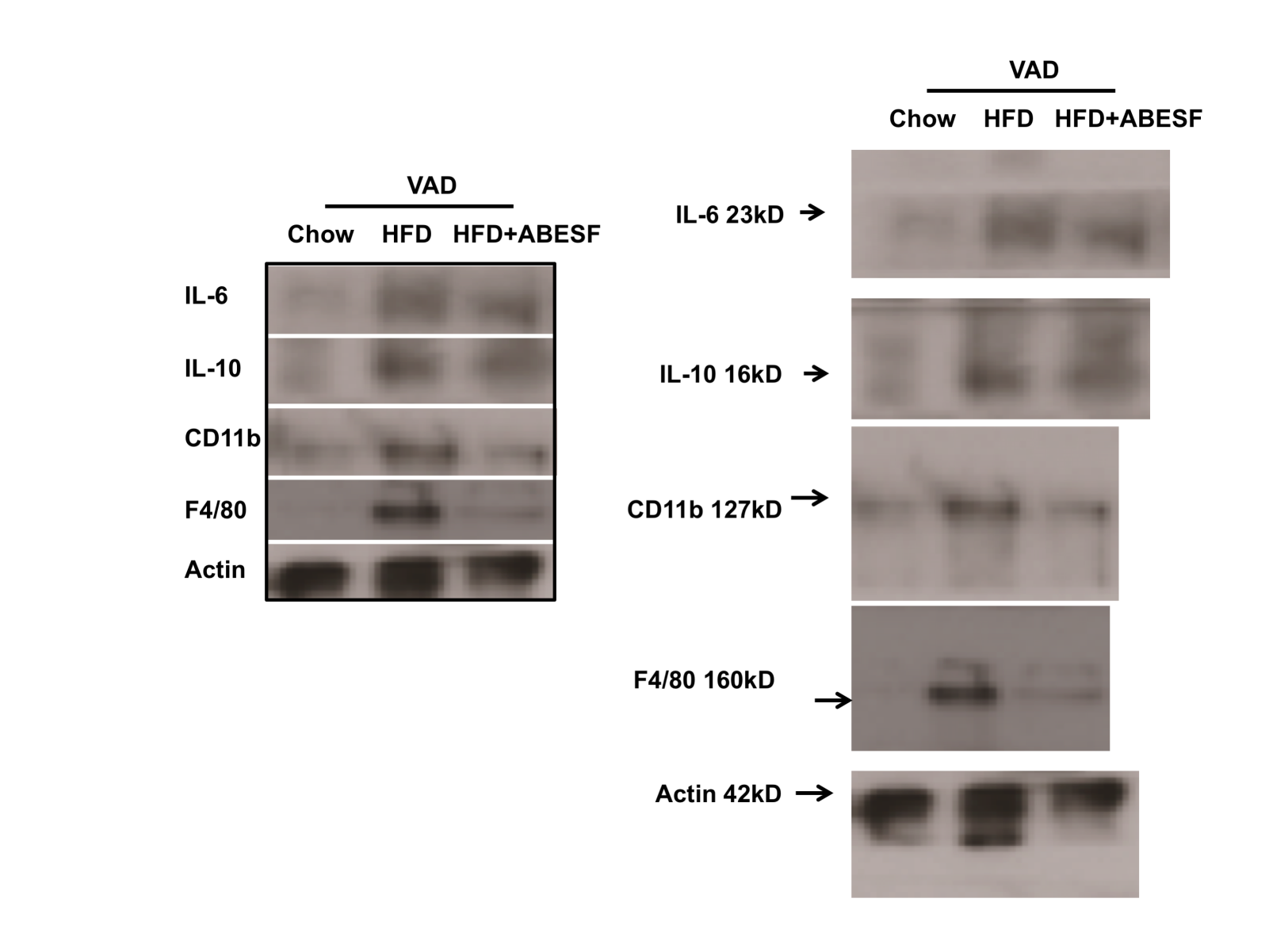
**
